# Retained Permanent Epicardial Pacemaker Leads and Lung Abscess: A Diagnostic and Surgical Challenge

**DOI:** 10.1016/j.jaccas.2025.106318

**Published:** 2025-12-03

**Authors:** Abdul Rasheed Bahar, Mohamad Hasan Jawadi, Shaun Cardozo, Kartik Kumar, Frank Alexander Baciewicz

**Affiliations:** a Department of Medicine, Wayne State University, Detroit, Michigan, USA; b Division of Cardiology, Department of Medicine, Wayne State University, Detroit, Michigan, USA; c Beth Israel Lahey-Winchester Hospital, Winchester, Massachusetts, USA; d Department of Cardiothoracic Surgery, Wayne State University, Detroit, Michigan, USA

**Keywords:** cardiac device infection, epicardial pacemaker leads, lead retention complications, pulmonary abscess

## Abstract

**Background:**

Epicardial pacemaker leads are used when transvenous access is contraindicated, but retained leads can rarely result in serious long-term complications.

**Case Summary:**

A 40-year-old male with intravenous drug use, tricuspid valve replacement, and prior epicardial pacemaker implantation presented with hemoptysis. Imaging revealed a left lower lobe lung abscess containing retained epicardial leads, left in situ after incomplete extraction years earlier. He underwent lobectomy and resection of infected leads. Postoperatively, he developed respiratory failure and arrhythmias requiring repeat epicardial pacemaker implantation, but ultimately recovered after rehabilitation.

**Discussion:**

Lung abscess due to retained permanent epicardial leads is exceedingly rare. This case underscores diagnostic challenges, the morbidity of incomplete lead removal, and the importance of vigilance in patients with prior cardiac device history.

**Take-Home Messages:**

Retained permanent epicardial leads are not biologically inert and may predispose to severe infectious complications. Complete hardware extraction and long-term surveillance are critical.

## Central Illustration

**Figure:**
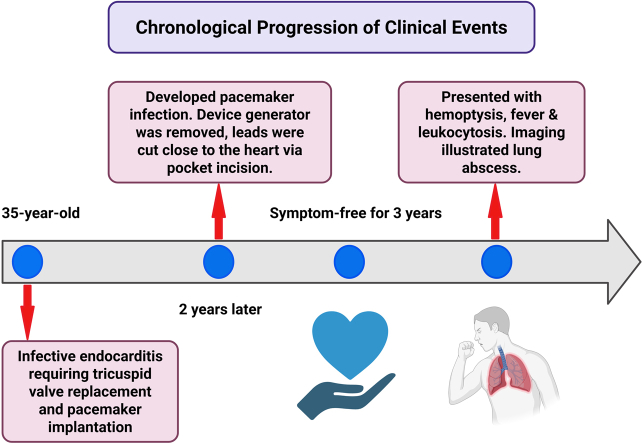

Cardiac implantable electronic devices (CIEDs), including pacemakers and implantable cardioverter-defibrillators, have revolutionized the management of bradyarrhythmias and heart failure. While endocardial lead systems are standard, epicardial lead placement is occasionally employed in patients with prosthetic tricuspid valves, congenital heart disease, or active infective endocarditis, where transvenous access may be contraindicated or anatomically challenging.^1^

The estimated risk of infection following initial CIED implantation is approximately 0.5% to 0.7%.^2^ However, this risk can be significantly elevated by factors related to the procedure, device type, and individual patient characteristics. Beyond contributing to substantial morbidity, CIED infections are also associated with increased mortality, with reported rates ranging from 4% to 13.7% and potentially exceeding 25% within the first year post infection.^3^

Although epicardial systems eliminate intravascular access, they are still prone to complications, primarily infections, with rarer adverse events including recurrent pericarditis, cardiac tamponade, and pericardial constriction. Incomplete extraction of epicardial leads, often due to technical limitations or dense adhesions, poses a unique challenge. Retained leads may serve as chronic foreign bodies, predisposing to late infectious complications and chronic inflammatory responses, including abscess formation.^4^

Lung abscesses from retained epicardial pacing wires are exceedingly rare and typically present years after implantation or failed extraction. Symptoms often mimic pulmonary malignancy or tuberculosis, particularly in high-risk patients, leading to frequent diagnostic delays. We report a left lower lobe abscess secondary to retained pacing wires in a patient with a remote history of tricuspid valve replacement for infective endocarditis.

## Case Presentation

A 40-year-old male with a history of intravenous drug use, chronic hepatitis C infection, tricuspid valve replacement secondary to *Staphylococcus aureus* endocarditis, and prior epicardial pacemaker implantation presented to the emergency department with complaints of hemoptysis that began 2 days prior. He reported blood-streaked yellow sputum with several clots along with fever but denied night sweats, weight loss, or shortness of breath.

**Figure 1:**
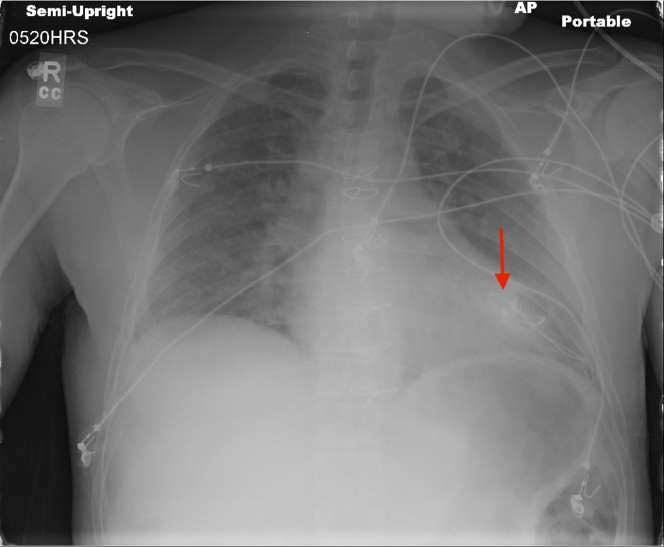
Chest X-Ray Demonstrating Retained Epicardial Pacing Leads (Arrow) Attached to the Heart and Adjacent to the Pericardium After Generator Removal

Five years earlier, the patient underwent tricuspid valve replacement and permanent epicardial pacemaker placement following an episode of right-sided infective endocarditis, due to the high risk of heart block after valve surgery and the avoidance of crossing a prosthetic valve with transvenous leads. Approximately 2 years after the implantation, the patient developed a pacemaker infection due to *S. aureus*. Although the generator was successfully removed, the complete extraction of the epicardial leads was unsuccessful. Because complete epicardial lead extraction would have required thoracotomy or sternotomy, efforts were made to remove as much of the leads as possible; however, only the generator and accessible portions were removed, and the remaining leads were ultimately transected as close to the heart as possible via the pocket incision and left in situ. Figure 1 illustrates a chest x-ray obtained after generator removal, demonstrating retained leads attached to the heart. He completed a full course of intravenous antibiotics with resolution of the clinically apparent pocket infection. However, the leads might have remained colonized, as is often the case in the setting of pocket infection. Consistent with Heart Rhythm Society guidance, we did not institute chronic suppressive antibiotics, as such therapy is generally not recommended for abandoned epicardial leads in the absence of ongoing clinical infection after adequate source control. The patient remained clinically stable for approximately 3 years (Visual Summary).

**Visual Summary:**
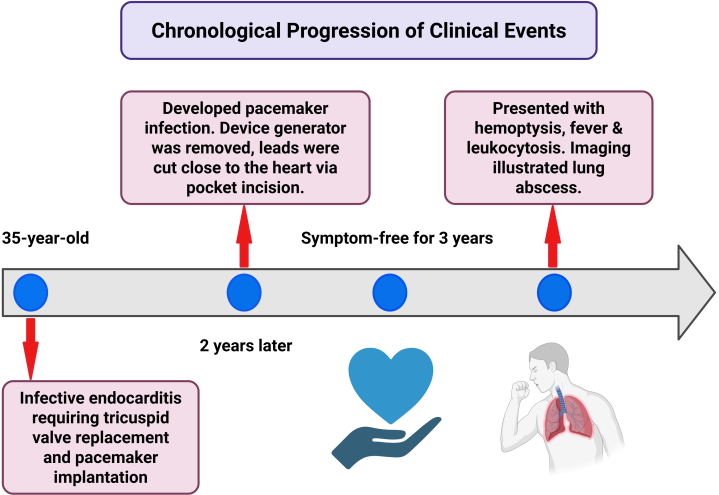
Timeline of Epicardial Lead Retention Leading to Delayed Lung Abscess

**Figure 2:**
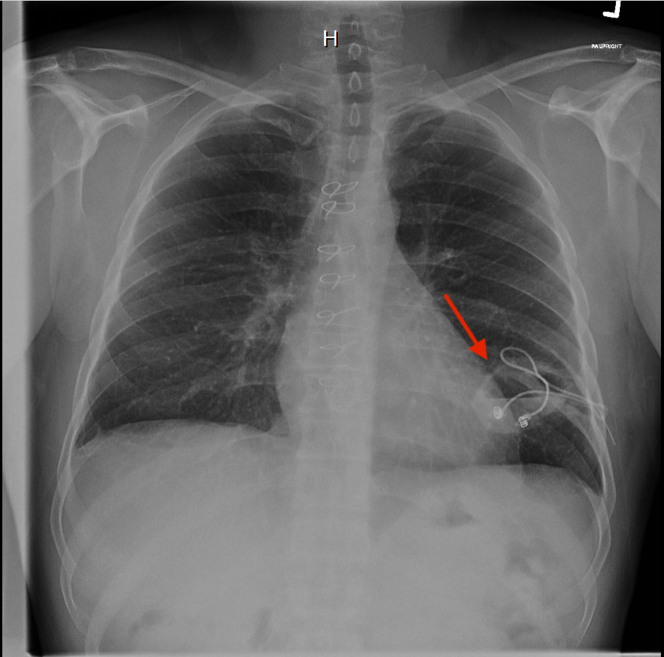
Chest X-Ray Illustrating a Left Lower Lobe Cavitary Lesion With Air-Fluid Level and Traversing Retained Epicardial Pacing Leads (Arrow)

**Figure 3:**
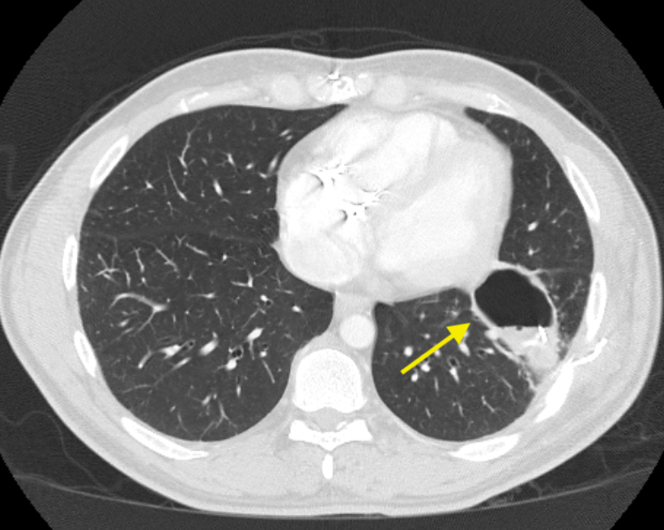
Chest CT Showing A Left Lower Lobe Cavitary Lesion With Retained Epicardial Leads (Arrow), Consistent With Pulmonary Abscess

On this admission, initial vital signs were within normal limits. Laboratory studies revealed leukocytosis (WBC 18.6 × 10^3^/μL) and normocytic anemia (Hb 8.6 g/dL). Initial chest radiography revealed a large cavitary lesion in the left lower lobe with an air-fluid level and visible pacing leads traversing the cavity (Figure 2). Chest computed tomography demonstrated a cavitary lesion (7 cm × 4 cm) in the left lower lobe containing retained epicardial pacemaker leads consistent with a pulmonary abscess (Figure 3). The patient was initiated on intravenous vancomycin and ceftriaxone. Blood and respiratory cultures were obtained, and both HIV and TB testing returned negative. Transthoracic echocardiography showed a bioprosthetic tricuspid valve with mildly elevated gradients, mild regurgitation, and no evidence of endocarditis.

The patient underwent left thoracotomy with total decortication, left lower lobe lobectomy, and mediastinal lymphadenectomy. Intraoperatively, dense adhesions were noted between the lung, chest wall, and pericardium. An abscess cavity containing pacemaker lead remnants was identified and resected. Tissue cultures grew methicillin-resistant *S. aureus*, prompting antibiotic de-escalation to intravenous clindamycin and subsequently ceftaroline.

Postoperatively, the patient developed acute hypoxic respiratory failure requiring re-intubation and tracheostomy, followed by Mobitz II AV block and atrial flutter. Permanent pacing was deferred until infection resolved, after which he underwent left parasternal thoracotomy for epicardial pacemaker re-implantation with repeat tracheostomy and percutaneous endoscopic gastrostomy placement. Neurocognitive recovery was complicated by postoperative agitation and delirium but improved with supportive care and adjusted psychotropic therapy. Due to persistent functional decline and deconditioning, he was discharged in stable condition to an acute inpatient rehabilitation facility for continued recovery. After a period of rehabilitation, the patient was discharged home in medically stable condition.

## Discussion

To the best of our knowledge, this represents the first documented case of a lung abscess resulting from migration of retained permanent epicardial pacemaker leads, distinguishing it from previously published reports that involved only temporary pacing wires. Epicardial pacing leads are broadly categorized into permanent and temporary types, each with distinct roles and risks. Epicardial lead placement is indicated when transvenous access is not feasible or safe such as in patients with mechanical tricuspid valves, venous occlusions, pediatric anatomy, or active infections and in certain surgical scenarios where epicardial access is readily available (Table 1).^5^

**Table 1:** Possible Indications for Epicardial Lead Placement

1.	Prosthetic tricuspid valve
2.	Unfavorable venous anatomy
3.	Poor sensing and impedance from tricuspid valve route
4.	Phrenic stimulation from tricuspid valve route
5.	Temporary pacing (eg, post cardiac surgery such as coronary artery bypass grafting)
6.	Pediatric population with congenital heart diseases
7.	Venous thrombus
8.	Recurrent bacteremia/Infective endocarditis

Temporary epicardial leads are used postoperatively for short-term rhythm monitoring and pacing support, most often after surgeries like coronary artery bypass grafting, valve surgeries, or operations for congenital heart disease.^6^ These wires are usually removed a few days after surgery; however, when retained, they may predispose to complications such as migration or infection. Temporary epicardial pacing wires have been reported to cause bronchial penetration, leading to endobronchial obstruction.^7^ Retained temporary epicardial leads have also been implicated in the development of retroaortic abscesses.^4^

Permanent epicardial leads are occasionally placed during cardiac surgeries such as valve repair, correction of congenital heart defects, or venous thrombus. While short-term retention is generally well-tolerated, long-term retention has been associated with various complications, including lead failure, elevated pacing thresholds, and infections. Rare but serious adverse events such as lead migration, skin erosion, abscess formation, and fistula development have also been reported.^8^ This case illustrates a rare but serious late complication of retained epicardial pacemaker leads, the development of a pulmonary abscess. Pulmonary abscess related to retained epicardial leads is exceedingly rare, with only isolated cases documented in the literature.^9^^,^^10^

Clinical presentations are often nonspecific, ranging from cough and hemoptysis to constitutional symptoms, which can mimic more common conditions like tuberculosis or malignancy, particularly in patients with risk factors such as intravenous drug use or incarceration. This diagnostic ambiguity frequently delays appropriate treatment and increases the risk of complications. Polomsky et al^9^ reported a rare case of epicardial pacemaker lead migration into the lung, which resulted in hemoptysis and empyema. Similar to our case, their patient presented with pulmonary complications years after lead implantation. The retained lead acted as a foreign body promoting infection, emphasizing the long-term risks associated with incomplete lead removal. However, unlike our case, their presentation primarily involved empyema and hemoptysis rather than abscess formation. Horng et al^10^ described progressive dyspnea after coronary artery bypass grafting due to retained temporary epicardial pacing wires causing an endobronchial obstruction with resultant bronchiectasis and recurrent postobstructive pneumonia. This case highlighted that retained epicardial wires can result in delayed respiratory symptoms and infections, consistent with the clinical timeline seen in our patient. Although abscess formation was not reported, the retained hardware led to significant morbidity, similar to our experience. Smith et al^4^ documented an unusual retroaortic abscess resulting from a retained epicardial pacing wire. This case underlined the potential for serious infectious complications involving retained pacemaker leads, with abscess formation being a critical feature. Notably, Smith's case involved unusual anatomic localization of the abscess, whereas in our patient, the abscess was localized to the left lower lobe of the lung.

In this case, the patient developed a pacemaker infection approximately 2 years after the initial device implantation. Although complete lead extraction is considered ideal in such scenarios, it was not pursued due to the anticipated need for thoracotomy; the leads were instead transected and left in situ. Unlike previously reported cases, our presentation is distinct in several key aspects. First, it involved the delayed development of a pulmonary abscess years after the initial device-related infection, underscoring the persistent long-term risks associated with retained epicardial leads. Second, the management was particularly complex, requiring lobectomy, tracheostomy, and eventual re-implantation of pacing hardware, highlighting the potential for substantial morbidity. Finally, imaging in this case was particularly illustrative, with retained lead fragments clearly visualized within the abscess cavity, a rare but diagnostically valuable finding not consistently documented in prior literature.

## Conclusions

This case reinforces that retained epicardial leads, though sometimes unavoidable due to surgical complexity or adhesions, are not biologically inert and may serve as chronic foci for infection. Complete hardware extraction should be pursued whenever feasible, particularly in younger or high-risk patients. Patients with retained leads require long-term clinical surveillance, especially if there is a prior history of device-related infection. Clinicians should maintain a high index of suspicion for hardware-associated complications in individuals presenting with unexplained pulmonary symptoms and a history of epicardial pacemaker implantation. Given the rarity but severity of such cases, this report adds to the growing evidence that incomplete lead removal can result in delayed, life-threatening complications, particularly in socially and medically vulnerable populations

**Take-Home Messages**

• Permanent epicardial leads, when left in situ, can serve as a nidus for serious late complications, including pulmonary abscess.
• Early consideration of complete hardware removal and structured follow-up is essential to prevent delayed morbidity..

## Funding Support and Author Disclosures

The authors have reported that they have no relationships relevant to the contents of this paper to disclose.
